# *Sargassum* Differentially Shapes the Microbiota Composition and Diversity at Coastal Tide Sites and Inland Storage Sites on Caribbean Islands

**DOI:** 10.3389/fmicb.2021.701155

**Published:** 2021-10-29

**Authors:** Vincent Hervé, Josie Lambourdière, Malika René-Trouillefou, Damien Alain Devault, Pascal Jean Lopez

**Affiliations:** ^1^Max Planck Institute for Terrestrial Microbiology, Marburg, Germany; ^2^Institut de Recherche sur la Biologie de l’Insecte, UMR 7261, CNRS—Université de Tours, Avenue Monge, Tours, France; ^3^Biologie des ORganismes et Ecosystèmes Aquatiques (BOREA), Muséum National d’Histoire Naturelle, Centre National de la Recherche Scientifique UMR-8067, Sorbonne Université, Institut de Recherche pour le Développement, Université de Caen Normandie, Université des Antilles, Paris, France

**Keywords:** macroalgae, methanogenic archaea (MA), sulfate-reducing bacteria (SRB), epibiont, microbial communities, protists, nematodes, *Sargassum*

## Abstract

Rafts of drifting pelagic *Sargassum* that are circulating across the Atlantic Ocean are complex ecosystems composed of a large number of associated species. Upon massive stranding, they lead to various socio-environmental issues including the inflow of contaminants and human health concerns. In this study, we used metabarcoding approaches to examine the differences in both the eukaryotic- and prokaryotic-associated communities from *Sargassum* present in two islands of the Lesser Antilles, namely Guadeloupe and Martinique. We detected significant differences in microbial community structure and composition between landing *Sargassum*, the surrounding seawater, and *Sargassum* from inland storage sites. In total we identified 22,214 prokaryotic and 17,679 eukaryotic OTUs. Among them, functional prediction analyses revealed a number of prokaryotes that might contribute to organic matter decomposition, nitrogen cycling and gas production, including sulfate-reducing bacteria at coastal landing sites, and methanogenic archaea at inland storage sites. We also found that Metazoan was the most abundant group in *Sargassum* samples, with nematode clades that presented exclusive or specific richness and abundance patterns depending on their *Sargassum* substrate. Together, these molecular inventories of the micro- and meiofauna communities provide baseline information for further characterization of trophic interactions, algal organic matter decomposition and nutrient transfers at coastal and inland storage sites.

## Introduction

Numerous seaweeds, including benthic and drifting pelagic *Sargassum* species, are becoming invasive in various regions of the world, creating potential threats to native species and local resources, and causing economic and health concerns (Hu and Fraser, 2016). Over the last decade, the whole Caribbean region and the west coast of Africa have been faced with massive tides of *Sargassum* (Wang et al., 2019). These holopelagic macroalgae shoals contains several morphotypes, with the most common one being *S. fluitans* and *S. natans* (Parr, 1939; Schell et al., 2015; Amaral-Zettler et al., 2017; Martin et al., 2021). The large amounts of seaweed biomass washed up along coastlines has direct and indirect consequences on beaches and on the functioning of nearshore ecosystems (Van Tussenbroek et al., 2017; Cabanillas-Teran et al., 2019; Rodriguez-Martinez et al., 2019), and a massive influence on the tourist industry and local economies (i.e., the cost of removing and disposing of piled-up *Sargassum*, the effect on housing markets, etc.). *Sargassum* tides can also have serious health impacts, not only because of the toxicity of the associated heavy metals and pollutants, but also through their decomposition which can lead to the production of hydrogen sulfide (H_2_S) and ammonia (NH_3_), which have been shown to cause a real threat to human health (Resiere et al., 2018).

Pelagic *Sargassum* in the Atlantic Ocean constitutes a floating ecosystem serving as a habitat for marine species like sea turtles, seabirds, fish or invertebrates. Additionally, a number of micro- and macro-epiphytes have been described (Parr, 1939; Weis, 1968; Fine, 1970; Casazza and Ross, 1972; Ryland, 1974; Haney, 1986; Jobe and Brooks, 2009; Ballard and Rakocinski, 2012; Witherington et al., 2012; Jacobucci and Leite, 2014; Susilowati et al., 2015; Monroy-Velazquez et al., 2019), and a few of them are endemic species. Endophytic and epiphytic communities of small eukaryotes are likely to be involved in the growth of *Sargassum*, and might contribute to nutrient uptake, macroalgae spore release and germination, the defense against bactericidal pathogens and other competing organisms, reproduction and settlement (Egan et al., 2013; Florez et al., 2017; Van Der Loos et al., 2019). In addition, exchanges between the *Sargassum* holobiont and the surrounding environment might have reciprocal impacts, and micro-organisms initially associated with algae growth might also contribute to their degradation processes (Florez et al., 2017). Indeed, microorganisms are likely to contribute to algae raft formation and maintenance, algae sinking and biodegradation upon beaching (De Fouw et al., 2016). In addition, the microbial communities that are associated with the seaweeds can thrive in new habitats, as postulated for the export of surface-dwelling fauna associated with *Sargassum* from the surface down to the seafloor (Baker et al., 2018).

At the molecular level, bacterial diversity has been investigated for floating *Sargassum* from various locations and for several species. Serebryakova et al. (2018) investigated the 16S rRNA gene sequences associated with the invasive benthic *S. muticum* from various locations in Portugal, and showed that the epibionts were dominated by Proteobacteria, Bacteroidetes, and Actinobacteria. The structure of the bacterial community has also been analyzed for drifting *S. horneri* from the Yellow Sea (Mei et al., 2019). As with other macroalgae studies, Mei et al. (2019) demonstrated differences at the genus level in the bacterial composition between the surrounding water and the drifting algae, and between drifting and nearshore algae Sea. Torralba et al. (2017) found that *Sargassum* complexes of two main surface drifting species (*S. natans* and *S. fluitans*), collected from the Gulf of Mexico, were associated with microbial communities dominated by *Rhodobacteraceae* and *Saprospiraceae*. Another molecular study on the bacterial community of drifting *Sargassum* has shown that the microbial profile can vary depending on location, abundance of zooplankton and nutrient levels (Michotey et al., 2020). Surprisingly, the composition of protists associated with *Sargassum* have been largely investigated using taxonomic and/or morphological analyses (Maples, 1984; Huffard et al., 2014; Damare, 2015; Coelho-Souza et al., 2017; Baker et al., 2018; Kim H. M. et al., 2019), but to our knowledge, only one study have described the molecular diversity of the eukaryotic plankton and epifauna associated with floating *S. horneri* (Kim H. M. et al., 2019).

It was expected that the physicochemical conditions encountered along the coasts, associated to different microbial communities in both the water column and beaches could induce large modification of the micro- and macrofauna associated with *Sargassum*. Moreover, the significant accumulation (*ca*., corresponding to a new food source) and decay of seaweed biological materials at landing sites are further factors that could largely influence the local biodiversity and the microbiota associated to *Sargassum*. The objective of the present study was to perform the first investigations of the microbial communities associated with *Sargassum* from coastal landing sites (i.e., surf zone and deposited on beaches), and from inland storage sites. We used metabarcoding approaches to describe both the prokaryotic and eukaryotic diversities from two islands of the Lesser Antilles, Guadeloupe and Martinique. By comparing molecular data from the seaweed-associated fractions, we hoped to identify microorganisms that are more specifically associated with stranding or more dried seaweeds. We also aimed to gain novel information on the diversity of the species that are involved in *Sargassum* aging and biodegradation.

## Materials and Methods

### Field Sampling Procedure

**FIGURE 1:**
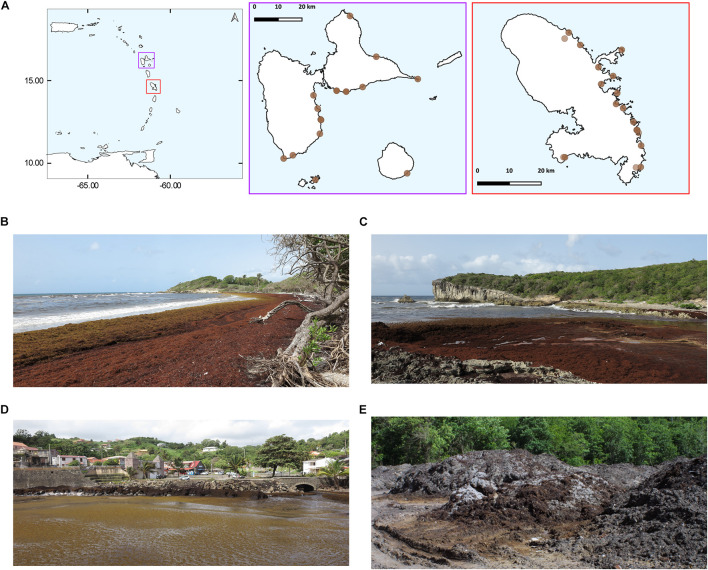
Maps of sampled *Sargassum* tide sites. The year 2018 corresponded to the biggest tide events ever recorded in the Caribbean region. **(A)** From left to right, the Caribbean Basin, the Guadeloupe archipelago (violet), and the island of Martinique (red). Due to the proximity of the sampled sites, some circles may overlap. **(B–E)** Photographs of sampling sites. *Sargassum* accumulated along the shorelines of Guadeloupe at Saint Félix **(B)**, and La Porte d’Enfer **(C)**. In Martinique, *Sargassum* in the small harbor of Le Marigot city **(D)**, *Sargassum* pileup at an inland storage site located in the township of Le Diamant **(E)**.

Various samples were taken along the Atlantic coasts of Martinique and Guadeloupe during the summer of 2018 (Figure 1). At coastal sites, for each sample, about 500 g of *Sargassum* seaweed were collected and the excess of seawater was gently drained off using a salad spinner, and then about 30 g were put into 50 ml Falcon tubes. At the same coastal landing sites, the surrounding seawater was also collected using sterile 50 ml Falcon tubes, at distances between 1 and 20 m from the shoreline and at about 0.5 m below the surface. In Martinique, we took samples from four different inland sites that were used by the local authorities to store the seaweeds collected on the beaches. For most of the tide sampling sites we collected at least one seawater marine sample and two *Sargassum* samples, and three samples for the inland storage sites. During the campaigns we placed all sample on ice, and then, upon arrival at the laboratory they frozen and maintained at –20°C until the nucleic acids were extracted.

### DNA Extraction and Library Preparation

The *Sargassum* samples were thawed at 4°C, suspended in 50 ml RNase-free water, vortexed and then mechanically sonicated in a water bath (35 kHz) for 3 min. The latter procedure was repeated three times. Then the microbial fractions present in the supernatant and extracted from the seaweed were filtered through a 0.2 μm GTTP Biofilm Type Filter kit (Millipore). About 50 ml of marine water samples were filtered using the same filter type that was mentioned above. The environmental DNA was extracted from the filters using the DNeasy PowerBiofilm Kit (Qiagen), in accordance with the manufacturer’s recommendations. Total DNA was then quantified using Quant-it PicoGreen (Invitrogen, Thermo Fischer Scientific, Carlsbad, CA, United States) and a CFX96 Real-Time System, C1000 Touch Thermal Cycler (Biorad, California, United States).

The DNA libraries were prepared using universal primers to target both prokaryotes and eukaryotes. To amplify the V4-V5 of the 16S rRNA, we used the 16S-515F-Y (5′-GTGYCAGCMGCCGCGGTAA-3′), and 16S-926R (5′-CCGYCAATTYMTTTRAGTTT-3′) primers that cover both bacteria and archaea (Walters et al., 2016). To amplify the V4 region of the 18S rRNA gene, we used the primer pair TAReuk454FWD1 (5′-CCAGCASCYGCGGTAATTCC-3′) and TAReukREV3 (5′-ACTTTCGTTCTTGATYRA-3′) (Stoeck et al., 2010). For each sample, we duplicated the initial DNA amplification in a final volume of 25 μl following the Taq Q5 Hot Start High Fidelity DNA Polymerase recommendations (New England Biolabs). For the 16S rRNA amplification, we also added to the amplification reactions 0.4 μl of a 10 μM of peptide nucleic acid (PNA) clamps (Fitzpatrick et al., 2018), that we designed to match 16S RNA genes from *Sargassum* plastid (pPNA: 5′-AGCTCAACTTCAAAACT-3′) and mitochondria (mPNA: 5′- GGCTAGCCTTATTCGTC-3′). For each sample, the PCR products were checked on agarose gels, purified using Agencourt AMPure XP beads (Beckman Coulter), and quantified using a Qubit dsDNA HS assay kit (Thermo Fisher Scientific). We then quantified and pooled the technical replicates per sample. To create unique library per amplicon type (16S or 18S), we pooled same quantity of all samples. These libraries were prepared using 1 μg pooled DNA and using the TruSeq Nano Library Preparation Kit (Illumina). The supplier’s protocol was followed, with the exception of the use of a modified End-Repair mix to avoid production of chimeric constructs, and no PCR cycle was done to finalize the libraries. The resulting libraries (16S or 18S) were quantified by qPCR and the paired-end (2 × 300 bp) sequencing were done using a HiSeq 2500 by Fasteris SA (Plan-les-Ouates, Switzerland).

### Sequence Processing

Amplicons of the 16S and 18S rRNA gene sequences were analyzed independently with the *mothur* software, version 1.41.3 (Schloss et al., 2009), following a standard operating procedure for Illumina MiSeq data (Kozich et al., 2013). First, contigs were assembled between the read pairs. Then the barcodes, primer sequences and low-quality sequences were removed (minimum length of 350 bp and maximum length of 420 bp, removing any sequences with ambiguous bases and any sequences with homopolymers longer than 8 bp). The sequences were then aligned with the SILVA reference database release 132 (Quast et al., 2013) and preclustered (*pre.cluster*, diffs = 1). Singletons were excluded, and chimeras were removed with *vsearch* (Rognes et al., 2016) implemented in *mothur*. The sequences were then classified using the k-nearest neighbor (*knn*) algorithm implemented in *mothur* and the BLASTN search method (cut-off of 80%) with the SILVA reference database, release 132, and the PR2 database, v4.11.1 (Guillou et al., 2013) for the 16S and 18S rRNA gene amplicons, respectively. After classification, non-prokaryotic, chloroplast, mitochondria (for the 16S rRNA gene dataset), non-eukaryotic, *Sargassum* (for the 18S rRNA gene dataset) and unknown (for both 16S and 18S rRNA amplicons) sequences were excluded. To account for differences in sampling efforts, 28,491 and 53,263 sequences from the 16S and 18S rRNA gene datasets, respectively, were then randomly subsampled from each sample using *mothur*. Finally, operational taxonomic units (OTUs) were generated using the *vsearch* distance-based greedy clustering algorithm, with an OTU being defined at the 97 and 99% sequence similarity level for the 16S and 18S rRNA gene reads, respectively. The raw datasets generated and analyzed during the current study were submitted to the NCBI Sequence Read Archive (SRA) under the BioProjects PRJNA630532 (16S rRNA genes) and PRJNA630533 (18S rRNA genes).

### Diversity and Statistical Analyses

*Alpha* diversity indices (observed OTU richness, Shannon and inverse of Simpson indexes) as well as Good’s coverage estimates and rarefaction curves were computed with *mothur*. All the statistical analyses were computed using R software version 3.6.3. To compare the *alpha* diversity indices between compartments, we used Kruskal-Wallis tests and corrected our *P*-values for multiple comparisons with the Benjamini-Hochberg method. Principal coordinates analyses (PCoA) and constrained analysis of principal coordinates (CAP) were computed based on Bray-Curtis dissimilarity matrices with the *phyloseq* package (McMurdie and Holmes, 2013). The effect of compartments on prokaryotic and eukaryotic community composition was tested by non-parametric permutational multivariate analysis of variance (PERMANOVA) on Bray-Curtis dissimilarity matrices, as implemented in the *vegan* function *adonis* (permutations = 9,999), after checking for homogeneity of group dispersions with the *vegan* function *betadisper* (Oksanen et al., 2015). We also used permutation tests for CAP, as implemented in the *vegan* function *anova.cca* (permutations = 9,999). To test the relationship between the prokaryotic and eukaryotic community matrices, a Mantel test was performed using the *ecodist* package (Goslee and Urban, 2007), with Pearson correlation coefficient and 10^6^ random permutations. To identify OTUs with differential abundances between compartments, we used the ALDEx2 algorithm on the prokaryotic and eukaryotic community matrices after filtering out the OTUs with less than 0.01% abundance (Fernandes et al., 2014). In this analysis, statistical significance was assessed based on the Wilcoxon rank test with Benjamini and Hochberg’s correction to maintain a 5% false discovery rate. Potential function among microbiota was predicted by using Functional Annotation of Prokaryotic Taxa (FAPROTAX v1.1), which was initially designed for marine samples (Louca et al., 2016). The predicted functional roles were visualized with the *pheatmap* R package, using Euclidean distances, and the weighted pair group method centroid (WPGMC) as a clustering method.

### Phylogenetic Analyses

Phylogenetic relationships between the abundant OTUs (>1% relative abundance) of Nematoda were investigated. We first collected nearly full-length 18S rRNA gene sequences from recent studies (Meldal et al., 2007) as reference sequences. These sequences were aligned with the Silva SSU database using SINA v1.2.11 (Pruesse et al., 2012) and the subsequent alignment was filtered using *trimAl* v1.2rev59 with the *gappyout* method (Capella-Gutierrez et al., 2009). OTU sequences were added to the reference alignment using the –*addfragments* option of MAFFT v7.310 (Katoh and Standley, 2013). The alignment was trimmed and Smart Model Selection (Lefort et al., 2017) was used to determine the best-fit evolutionary model based on the Akaike Information criterion. Subsequently, a maximum-likelihood phylogenetic tree was built with PhyML 3.0 (Guindon et al., 2010). Branch supports were calculated using a Chi2-based parametric approximate likelihood-ratio test (aLRT) (Anisimova and Gascuel, 2006). Finally, the tree was visualized and edited with iTOL (Letunic and Bork, 2019).

## Results

### Sampling

In the present study, we investigated both the prokaryotic and eukaryotic communities associated with *Sargassum* at landing sites along the Atlantic coastlines of the Guadeloupe archipelago and the island of Martinique (Figure 1). We analyzed seawater surrounding *Sargassum* from different tide sites (*n* = 52; 34 sites). Marine *Sargassum* samples were collected either in water (30 samples; 27 sites) or stranded on beaches (9 samples; 6 sites). Unfortunately, we could not obtain any specific information on the arrival dates of the *Sargassum* and/or on landing duration. Such data are in fact very difficult to obtain also because the local parameters (i.e., currents, wind, tide, etc.) and sinking processes can vary from place to place. Nevertheless, measurements on 87% of the collected samples revealed that the dry matter content was 16.2% ± 1.8%, showing that all samples had a similar hydration level (not shown). In Martinique, we also collected samples from inland storage sites (9 samples; 4 sites). Since there was no available information on the deposition period, we took samples from the middle and the top of the piles; partial measurements indicated that the *Sargassum* dry matter (2 sites) was 49.9% (not shown). As mentioned above because we analyzed seaweed samples that were fresh, partially or dried we did not and often could not separate the morphotypes that composed *Sargassum* samples. Hereafter, the three types of delineated biotic entities, hereafter named compartments, correspond to seawater from tide sites (TS-sw), *Sargassum* from tide sites (TS-sarg), and *Sargassum* from inland storage sites (ISS-sarg).

### Overall Diversity and Community Structure

After sequencing of the 100 samples and following bioinformatic procedures, we obtained a total of 22,214 prokaryotic and 17,679 eukaryotic operational taxonomic units (OTUs). For both datasets, the rarefaction curves (Supplementary Figure 1) as well as the high Good’s coverage estimates (median value 0.977 for the prokaryotes, Supplementary Figure 2; median value 0.997 for the eukaryotes Supplementary Figure 3) indicated that the sequencing depths were sufficient to estimate and compare the microbial diversity of our samples.

Regarding the *alpha* diversity of the prokaryotes, we found no significant differences in OTU richness nor diversity between the three compartments (*P* > 0.05) (Supplementary Figure 2). For the eukaryotes, we found significantly more OTUs in the seawater (TS-sw) than in the terrestrial and marine *Sargassum* samples (*P* < 0.001) (Supplementary Figure 3). For both the Shannon index and the inverse of Simpson index, eukaryotic diversity was significantly higher in TS-sw than in TS-sarg (*P* < 0.001) but no differences were found between TS-sarg and ISS-sarg (*P* > 0.05) (Supplementary Figure 3).

**FIGURE 2:**
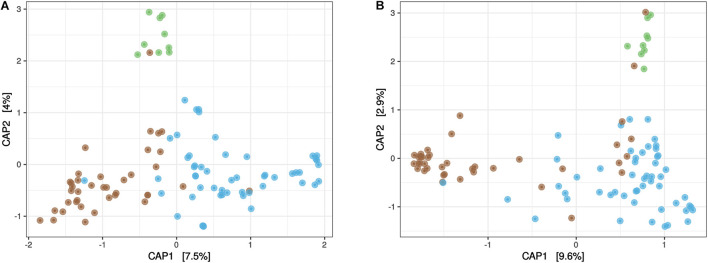
Differences in community composition. Both the prokaryotic community **(A)** and eukaryotic communities **(B)** presented significant differences (permutation tests for CAP, *P* < 0.001) between seawater at tide sites (TS-sw, in blue), *Sargassum* at marine tide sites (TS-sarg, in brown), and *Sargassum* at inland storage sites (ISS-sarg, in green).

*Beta* diversity of the *Sargassum* microbiota was first visualized using unconstrained ordinations, namely principal coordinates analyses (PCoA) (Supplementary Figure 4) focusing on the three compartments (TS-sw, TS-sarg, and ISS-sarg). PERMANOVAs indicated that both the prokaryotic (*R*^2^ = 0.08, *P* < 0.001) and the eukaryotic (*R*^2^ = 0.09, *P* < 0.001) communities were significantly different across the three compartments TS-sw, TS-sarg, and ISS-sarg. Differences between compartments were further investigated using constrained ordinations, namely canonical analysis of principal coordinates (CAP) (Figure 2), and confirmed by permutation tests for CAP (*P* < 0.001). For both the prokaryotic and the eukaryotic communities, the first axis discriminated between TS-sarg and TS-sw, while the second axis discriminated between ISS-sarg and both the TS-sarg and TS-sw samples (Figure 2). Among the TS-sarg samples, we found no differences between samples collected in the water or on the beaches (ANOSIM, *R* = –0.07, *P* = 0.78 and *R* = 0.01, *P* = 0.42, for the prokaryotic and eukaryotic communities, respectively). We also examined the relationship between the prokaryotic and eukaryotic dissimilarity matrices. A Mantel test revealed that the community structures of prokaryotes and eukaryotes were strongly correlated (*r*_*M*_ = 0.43, *P* < 0.0001), indicating potential biotic associations between microbiota.

### Diversity of the *Sargassum*-Associated Prokaryotic Communities

**FIGURE 3:**
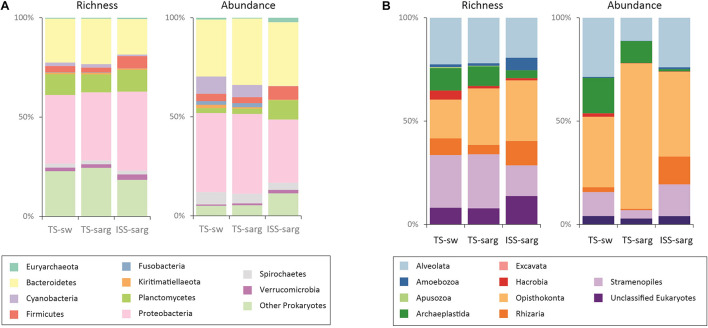
Molecular inventory of the biodiversity associated with *Sargassum* tides from 100 sampled sites. Differences in the observed OTU richness and relative abundance of seawater at tide sites (TS-sw), *Sargassum* at tide sites (TS-sarg) and inland *Sargassum* from storage sites (ISS-sarg). **(A)** Prokaryotic diversity corresponds to 22,214 OTUs obtained from 2,849,100 reads. **(B)** Eukaryotic diversity corresponds to 17,679 OTUs obtained from 5,326,300 reads.

The overall prokaryotic diversity corresponded to 658 bacterial and archaeal families distributed across 350 orders, and 57 phyla. For the TS-sarg samples we found that the most diverse phyla were Proteobacteria (34.3% of the richness in this compartment), Bacteroidetes (22.9%), and Planctomycetes (9.0%) (Figure 3A, also see Supplementary Figure 5A). At the phylum taxonomic rank, the diversity pattern present similarities with the TS-sw compartment, but differences were observed for the ISS-sarg samples (Figure 3A, and Supplementary Figure 5). Differences in diversity pattern of the three compartments were visible when analyzed at lower taxonomic ranks, with differences in the ten most rich orders (Supplementary Figure 6) or families (Supplementary Figure 7).

In the samples from coastal tide sites (TS-sw and TS-sarg), the most abundant phyla were the Proteobacteria, followed by Bacteroidetes, Cyanobacteria, Spirochaetes and Firmicutes. For the inland storage sites (ISS-sarg), the most abundant phyla corresponded to Bacteroidetes, Proteobacteria, Planctomycetes, Firmicutes and Acidobacteria (Figure 3, also see Supplementary Figure 5B). In fact, differences in relative abundances between the three compartments and specially the terrestrial one, were discernible at all taxonomic levels analyzed, as shown at the order (Supplementary Figure 6) and family (Supplementary Figure 7) levels.

Even though there were differences in the number of samples per compartment, we analyzed the number of OTUs shared between them. The Venn diagram showed that 9.3% of the richness was shared between the three compartments (Supplementary Figure 8), corresponding to members of 4 archaeal and 41 bacterial phyla.

### Diversity of the *Sargassum*-Associated Eukaryotic Communities

The eukaryotic diversity spread across 278 orders, with an overall dominance of Opisthokonta in terms of both richness (24.9%) and read abundance (48.9%), followed by the Alveolata (22.3% of richness and 21.4% of relative abundance), and the Stramenopiles (20.7% of richness and 9% of relative abundance). Independent analyses of the three compartments revealed differences in both OTU richness and relative abundance (Figure 3B, and Supplementary Figure 5). We found that 2.2% of the eukaryotic OTUs were present in all three compartments, a result which was lower than the one obtained for the prokaryotes (Supplementary Figure 8).

Specially in TS-sw samples we found that the phototrophs were among the most abundant organisms with key groups corresponding to Bacilliophyta, Florideophyceae, Ulvophyceae and Dinophyceae (Supplementary Figures 9, 10). In fact, in both kind of tide site samples (TS-sw and TS-sarg), the phylum that presented the highest richness were the Bacilliophyta (Supplementary Figure 9), with highest abundance of polar centric and raphid pennate diatoms in TS-sw and TS-sarg, respectively (Supplementary Figure 10). Compared to the tide site samples, differences in both richness and relative abundance were visible at all taxonomic ranks for the ISS-sarg ones (Figure 3B and Supplementary Figures 5–10).

**FIGURE 4:**
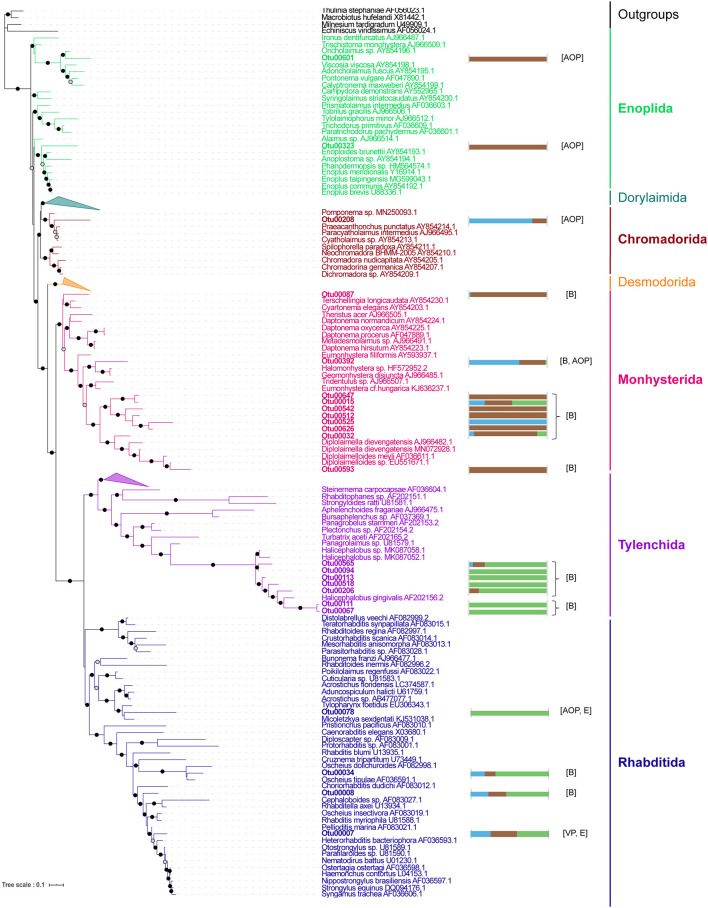
Phylogenetic relationships between the most abundant nematode OTUs associated with *Sargassum*. The maximum likelihood phylogenetic tree was constructed with PhyML v3.0 using the GTR + G + I model. Branch supports were calculated using a Chi2-based parametric approximate likelihood-ratio test and are represented by black circles for values above 0.90 and gray circles for values between 0.7 and 0.89. OTU sequences are in bold. The horizontal bar plots represent the normalized total abundance of each OTU in the different compartments: TS-sw in blue, TS-sarg in brown, and ISS-sarg in green. Putative trophic groups were assigned following recommendations by Meldal et al. (2007): algivore-omnivore-predator (AOP), bacterivore (B), entomopathogen (E), and vertebrate parasite (VP).

Focusing on the Metazoa, we found strong differences between the compartments, with the Bryozoa dominating the TS-sarg samples (Supplementary Figure 11). A large abundance of Bryozoans associated with *Sargassum* (42% of average relative abundance for the TS-sarg samples) was expected because of the large number of them visible on axe, blade, receptacle and vesicle surfaces (see Supplementary Figure 12). Because the Nematoda were the most dominant group in terrestrial samples (Supplementary Figure 11), the taxonomy of the most abundant OTUs was refined by phylogenetic analysis and their putative membership to trophic groups were inferred following Meldal et al.’s (2007) classification (Figure 4). We identified 24 OTUs belonging to five orders, namely Enoplida, Chromadorida, Monhysterida, Tylenchida, and Rhabditida. Among the Monhysterida, ten OTUs including seven potentially bacterivorous were particularly abundant in TS-sarg. Conversely, seven Tylenchida OTUs, all assigned to the genus *Halicephalobus*, and the four Rhabditida OTUs were mainly present in the ISS-sarg samples. Besides bacterivores, we identified nematodes that are related to species known to be algivores-omnivores-predators, entomopathogens and vertebrate parasites (Figure 4).

### Identification of Discriminant Operational Taxonomic Units for Each Compartment

**FIGURE 5:**
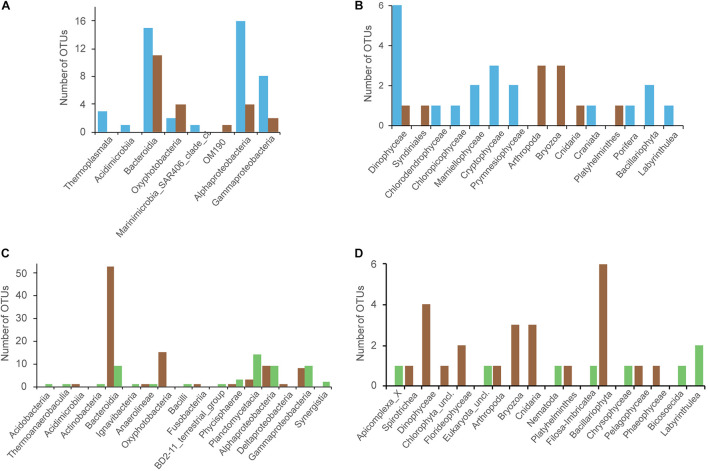
Identification of the OTUs presenting significant differential abundance for the different *Sargassum*-associated samples, *via* the ANOVA-like differential expression (ALDEx2) analysis. Comparison between tide sites OTUs that are differentially represented between seawater (TS-sw, *blue*) or *Sargassum* (TS-sarg, *brown*): **(A)** Prokaryotic, and **(B)** Eukaryotic. Analyses of the *Sargassum*-associated OTUs that are differentially represented between marine *Sargassum* (TS-sarg, *brown*) or terrestrial *Sargassum* (ISS-sarg, *green*): **(C)** Prokaryotic, and **(D)** Eukaryotic.

Because we demonstrated significant differences in community structure between the three compartment (Figure 2), we then investigated the OTUs that present differential abundance using ALDEx2 algorithm. We first made comparisons between the surrounding seawater and *Sargassum* at tide sites. We identified 68 prokaryotic and 30 eukaryotic OTUs with differential abundance (Figures 5A,B and Supplementary Table 2). For the prokaryotes, the TS-sw samples presented 46 OTUs that were significantly more abundant than in the TS-sarg samples (Figure 5A, blue bars). On the other hand, we found 22 OTUs preferentially associated with the TS-sarg samples compared to the seawater (Figure 5A, brown bars). In the case of the eukaryotes, the 20 OTUs were significantly more abundant with the TS-sw seawater compartment, and 10 OTUs for the TS-sarg one (Figure 5B).

When comparing the communities associated to *Sargassum* collected at tide sites compared to *Sargassum* from inland storage sites, we identified 146 prokaryotic and 32 eukaryotic OTUs with differential abundance (Figures 5C,D and Supplementary Table 2). Coastal *Sargassum* samples showed a significant enrichment of 93 OTUs prokaryotic OTUs, including members of the *Saprospiraceae*, *Flavobacteriaceae*, *Cyanobacteria*, *Alphaproteobacteria*, and *Gammaproteobacteria* (Figure 5C). The prokaryotes found to be enriched in terrestrial samples represented 53 OTUs distributed over 13 classes (Figure 5C). For the eukaryotes, the marine *Sargassum* showed a significant enrichment of 24 OTUs, among them we found Dynophyceae, Bryozoans, Hydrozoans, or Bacillariophyceae (Figure 5D). The *Sargassum* samples from storage sites showed a significant enrichment of 8 OTUs, including one nematode (Figure 5D, green bars).

### Functional Inference of Prokaryotic Diversity

**FIGURE 6:**
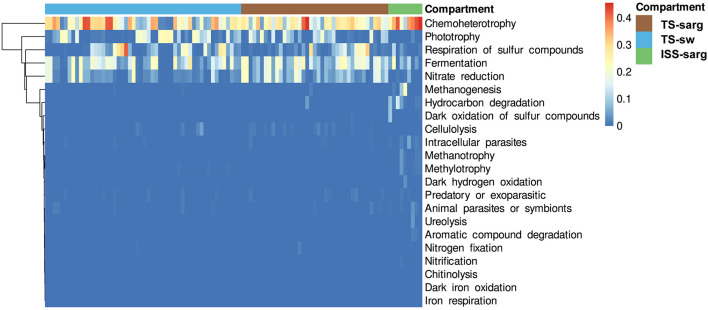
Heatmap of predicted functions based on the FAPROTAX database. The 22 categories presented here correspond to 2,831 prokaryotic OTUs. Functions were clustered with the weighted pair group method centroid (WPGMC) algorithm based on Euclidean distances. The color scale represents the proportion of each predicted function within a sample.

To investigate the putative roles of the prokaryotic taxa associated with the *Sargassum* samples, we used FAPROTAX and obtained putative functional assignment for 2,831 prokaryotic OTUs (12.7% of the global dataset) distributed into a minimum set of 22 functional groups (Figure 6 and Supplementary Table 3). The five most abundant ones corresponded to chemoheterotrophy (1,260 OTUs; 23.19% of the relative abundance), fermentation (314 OTUs; 13.04% of the relative abundance), nitrate reduction (71 OTUs; 9.32% of the relative abundance), phototrophy (388 OTUs; 6.98% of the relative abundance), and respiration of sulfur compounds (404 OTUs; 3.91% of the relative abundance) (Figure 6). Intracellular parasites, predators or exoparasites, and animal parasites or symbionts, corresponded to a relatively large number of organisms with 491, 263 and 189 OTUs, respectively. Interestingly, other functional groups specifically abundant in terrestrial samples corresponded to methanogenesis (32 OTUs; 0.21%), hydrocarbon degradation (21 OTUs; 0.31%), and dark oxidation of sulfur compounds (20 OTUs; 0.09%).

## Discussion

*Sargassum* washout in the coast of the Caribbean region, including the Gulf of Mexico, and in the coasts of Africa are causing many environmental and health concerns. Part of these concerns are probably the results of the load of new microbes associated with the seaweed, and the putative transient or persistent modifications of the microbial communities at tide sites. Here we investigated samples from three different compartments, namely from seawater and *Sargassum* from tide sites (TS-sw and TS-sarg samples, respectively) and *Sargassum* from inland storage sites (ISS-sarg samples). We found that the *alpha* diversity was similar for prokaryotes in the three compartments, and that the richness was higher for eukaryotes in seawater compared to the *Sargassum*-related samples. *Beta* diversity analyses revealed clear distinctions between the three compartments and proved a correlation between eukaryotic and prokaryotic communities. When analyzed at the high taxonomic ranks, compositional analyses revealed some similarities between the *Sargassum* and the surrounding water, even if differences exist at lower taxonomic ranks, also see below. This result somehow contrasted with previous reports on *Sargassum* (Torralba et al., 2017; Michotey et al., 2020), but those corresponded to analyses of *Sargassum* that were collected in offshore regions. In fact, beside the well documented technical biases when comparing samples across studies (DNA extraction, primer choice, PCR program, sequencing platform, etc.), we believe that differences between published data on the *Sargassum* microbiota could be due to a number ecological reasons including spatial and temporal variability in *Sargassum*-associated communities (Huffard et al., 2014; Torralba et al., 2017; Serebryakova et al., 2018; Mei et al., 2019; Michotey et al., 2020), biogeochemical factors at tide sites compared to offshore, or aging and drowning of the seaweeds (Baker et al., 2018; Sanders-Smith et al., 2020).

### *Sargassum*-Associated Prokaryotic Communities, and Their Putative Environmental Roles

The most abundant prokaryotic family associated with the *Sargassum* samples from tide sites (TS-sarg) was the *Vibrionaceae* (Proteobacteria) (*ca*. 18% of the relative abundance, see Supplementary Figure 7), a family of ubiquitous marine bacteria. The second most abundant family was the *Flavobacteriaceae* (Bacteroidetes). Interestingly, utilization of macromolecules such as polysaccharides is a common feature of many members of this family (McBride, 2014; Thomas et al., 2021), and more generally of the Bacteroidetes (McKee et al., 2021). In fact, several genera of the *Flavobacteriaceae* that were found to possess conserved polysaccharide utilization loci, as defined by Kruger et al. (2019), were abundant at tide sites, with the NS5_marine_group, *Polaribacter* spp., *Formosa* spp., *Dokdonia* spp., *Gramella* spp., *Cellulophaga* spp., *Lutibacter* spp., *Winogradskyella* spp., *Cryomorphaceae*, *Crocinitomicaceae*, and *Cyclobacteriaceae* together making up to 12.9 and 4.3% of the relative abundance in TS-sw and TS-sarg samples, respectively. These results suggest that these bacteria might be involved in the degradation of polysaccharides either exudate or release by the seaweeds and the associated epifauna, and probably contribute to *Sargassum* degradation. The third most abundant family was the *Saprospiraceae* (Bacteroidetes), another bacterial clade harboring species capable of degrading complex macromolecules produced by seaweeds and their associated algae (Mcilroy and Nielsen, 2014). The *Flavobacteriaceae* (Bacteroidetes) family was the most abundant for terrestrial storage sites (Supplementary Figure 7), again suggesting a possible contribution of some of these bacteria to organic matter decay. Further studies should address the shift in microbial communities in the water column of coastal areas, depending on the *Sargassum* morphotypes, algal biomass and aging.

Using FAPROTAX, we obtained functional prediction for a number of prokaryotic OTUs. We found that the most abundant ones corresponded to chemoheterotrophy, nitrate reduction, phototrophy, and respiration of sulfur compounds. Interestingly, putative intracellular parasites, predators or exoparasites, and animal parasites or symbionts, corresponded to a relatively large number of organisms. Within terrestrial samples, these functional predictions identified abundant OTUs (2.0% of the relative abundance in ISS-sarg) potentially involved in methanogenesis, and corresponding to euryarchaeota assigned to *Methanomicrobiaceae*, *Methanosarcinaceae*, and *Methanococcaceae*. Members of these families have been reported to be involved in methane production from algal biomass (Bapteste et al., 2005; Adam et al., 2017; Klassen et al., 2017). Our results suggest that at inland storage sites, a portion of the carbohydrates from the seaweed alga biomass (for *Sargassum* see Devault et al., 2021, and references therein) is probably degraded anaerobically by these archaea, and used as potential energy source (Smetacek and Zingone, 2013; Helbert, 2017; Milledge et al., 2020).

When washed ashore, *Sargassum* decomposition processes can lead to hydrogen sulfide (H_2_S) and ammonia (NH_3_) gas emissions, which can result in significant environmental issues (Van Tussenbroek et al., 2017; Cabanillas-Teran et al., 2019; Rodriguez-Martinez et al., 2019) and health concerns (Resiere et al., 2018, 2020). As mentioned above, functional prediction identified OTUs involved in the respiration of sulfur compounds (Figure 6), which represent 3.8, 4.7, and 0.9% of the relative abundance in TS-sarg, TS-sarg and ISS-sarg compartment, respectively. These OTUs were assigned to several orders including *Desulfobacterales* (257 OTUs), *Desulfovibrionales* (87 OTUs), *Clostridiales* (51 OTUs), *Desulfarculales* (35 OTUs), *Desulfuromonadales* (3 OTUs), *Syntrophobacterales* (3 OTUs), and *Thermococcales* (1 OTU). Member of these orders, except the *Thermococcales* were present in the three compartments. In addition to the FAPROTAX predictions, we found in the three compartments additional OTUs assigned to *Sulfurovum* and *Sulfurimonas* (*Campylobacterales*) which are genera known to be involved in sulfur oxidation, and thus probably also contribute to the sulfur cycle in our samples (Han and Perner, 2015). A deeper understanding of the diversity of sulfate-reducing and sulfur-oxidizing microorganisms associated with *Sargassum* decomposition processes will require specific investigations, such as the presence of functional sulfur cycling-related genes (Pelikan et al., 2016). Furthermore, it will be important to further link the sulfur cycle to several others such as carbon, metals and metalloids such as arsenic (As) (Fisher et al., 2008; Edwardson and Hollibaugh, 2017). This issue seems particularly relevant to us given the large amount of arsenic that bioaccumulates in various *Sargassum* species (see Rodriguez-Martinez et al., 2020; Devault et al., 2021, and our unpublished data for Guadeloupe and Martinique).

Nitrate reduction is another predicted functional role that might be played by prokaryotes. The 71 OTUs functionally predicted to contribute to nitrate reduction belong to 16 bacterial families (Supplementary Table 3) with the most abundant OTUs assigned to the genus *Vibrio* (4.6 and 17.4% of the relative abundance in TS-sw and TS-sarg). This genus, was found to present variable and eventually very high abundance in pelagic *Sargassum* samples (Michotey et al., 2020), could contribute to nitrogen cycling (Urdaci et al., 1988; Herbert, 1999) and potentially to the host nitrogen metabolism. *Vibrios* have been shown to also contribute to the marine organic carbon cycle (Martin et al., 2015; Zhang et al., 2018), and to potential sanitary risks because many of them are pathogenic to animals or humans (Baker-Austin et al., 2017). Regarding the nitrogen cycle, we also found 12 prokaryotic OTUs potentially involved in nitrogen fixation as well as 19 OTUs potentially involved in nitrification. Recent analyses have shown that %N has increased over the years in *Sargassum* and that this percentage is higher in *Sargassum* from coastal waters (see Lapointe et al., 2021 and references therein) raising the importance to further study N metabolism of landing *Sargassum*.

### The Metazoa Are the Most Abundant Eukaryotes Associated to *Sargassum* Samples

The eukaryotic community associated to *Sargassum* has been rarely investigated by molecular approaches (Kim H. M. et al., 2019). Here we demonstrate that the eukaryotic communities of *Sargassum* samples is dominated by Metazoa, with 24.8% of diversity and 69.9% of the relative abundance from tide sites, and 20.3% of diversity and 38.0% of the relative abundance for the inland storage sites. We expected that some of them correspond to the non-motile and motile epifauna species (Stoner and Greening, 1984; Martinsmith, 1994; Gestoso et al., 2012; Martin et al., 2021). Indeed, crustacea were the most abundant clade in the TS-sw and the second most abundant in the TS-sarg samples, and corresponded essentially to the class Maxillopoda. Copepods (Maxillopoda) have already been described as an abundant invertebrate fauna associated with floating *Sargassum* (Ólafsson et al., 2001; Abé et al., 2013). Among the 555 Maxillopoda OTUs found from the tide site samples, 16.4% were shared between the seawater (TS-sw) and *Sargassum* (TS-sarg) samples, and 36.9% were exclusive to TS-sarg. The most abundant copepod that was found to be shared between these two compartments was assigned to the genus *Zaus* (best blast hit), a genus already found to be associated with macroalgae (Kangtia et al., 2015). We hypothesized that colonization of *Sargassum* by copepods could have occurred during rafting, explaining why different communities could be found between *Sargassum* and seawater at tide sites. Alternatively, some of the copepods identified might correspond to genuine coastal species, and that for some of them the arrival of *Sargassum* and macroalgal detritus could have correspond to new ecological niches to colonize.

Association between nematodes and floating *Sargassum* in the Atlantic region was already noted a century ago (Micoletzky, 1922). The colonization of such usually benthic meiofauna could be the result of accidental encounters and dispersal when clumps of algae are carried by wind or currents. Indeed, benthic nematode species were previously found attached to the surface of pelagic or benthic *Sargassum* spp. (Gerlach, 1977; Kito, 1982; Vranken et al., 1986; Kim H. G. et al., 2019). Within our dataset, we specifically investigated the most abundant nematode-related OTUs and, interestingly, found specific patterns depending on the substrate (Figure 4). The nematodes that were found to be associated with *Sargassum* at tide sites belong to the Enoplia and Monhysterida clades that have been described in marine habitats. The trophic behaviors of species from these clades have been shown to correspond to bacterivore and algivore-omnivore-predator (Meldal et al., 2007; Smythe, 2015). Nematodes were largely dominant in terrestrial samples, with 92% of the metazoan’s read abundance. OTUs assigned to Tylenchina, which corresponds mostly to plants, invertebrate and vertebrate parasites, and to Rhabditina, which are generally terrestrial free-living nematode species (Meldal et al., 2007), showed exclusive or specific patterns with inland *Sargassum* samples (Figure 4). Regarding the OTUs related to *Halicephalobus*, the best blast hit for Otu00067 and Otu00111 was *Halicephalobus* sp. (accession number GQ918144), a bacterivore nematode that has been found in the terrestrial deep subsurface in hypoxic conditions (Borgonie et al., 2011). The hypoxic conditions within the piles of *Sargassum* at storage sites are likely to be one of the important factors explaining differences in the distribution of nematode species within compartments.

### Identification of *Sargassum* Specific Operational Taxonomic Unit Associations

In the present study we also identified OTUs that presented differential abundance at tide sites, or between coastal and terrestrial *Sargassum* samples. In total, we found 199 prokaryotic and 56 eukaryotic OTUs with differential abundance in our compartments (Supplementary Table 2). At tide sites, the eukaryotic OTUs significantly more abundant in the surrounding water corresponded to Dinophyceae, unclassified Maxillipoda, Bryozoa [probably to the genus *Membranipora*, a hydrozoan of the genus *Zanclea* that was previously described as being common on a hydroid assemblage on holopelagic *Sargassum* from the Sargasso Sea (Calder, 1995)], and one OTU of flatworm of the Rhabdocoela order. Some of these later species are known to live in association with *Sargassum* (Willems et al., 2004). Among the 24 OTUs that were significantly associated with the TS-sarg samples, we found one OTU assigned to a toxic dinoflagellate of the *Amphidinium carterae* species, another to *Ulvella* that are endophytic microalgae, one related to the raphid diatom genus *Aneumastus*, one OTU with the best BLASTN results as *Sargassococcus simulans*, an epiphyte on floating *Sargassum thallus*, isolated in the Sargasso Sea, one to the copepod genus *Zaus*, which are organisms living in the phytal zone, and five OTUs belonging to Hydrozoans, which are also frequently encountered as *Sargassum* epibionts (Huffard et al., 2014). The *Sargassum* samples from storage sites showed significant enrichment of 8 OTUs, including a flagellated protist of the Colpodellidae order, a nematode assigned to the Monhysterida order, and a filose amoeba.

Among the prokaryotes differentially abundant in *Sargassum* from tide sites, we identified several *Oxyphotobacteria* and *Saprospiraceae* which have often been reported as associated with macroalgae surface (Florez et al., 2017). Members of these family were also differentially abundant when comparing *Sargassum* samples between tide sites and inland storage sites. In particular, we found at tide sites 3 *Rubinisphaeraceae* OTUs preferentially associated with inland storage sites, and this family of Planctomycetes was also recently found in wastewater treatment plants (Al Ali et al., 2020), a putatively new member of the *Rivularia*-like cyanobacterium, and members of *Rhizobiales* Altogether, our data revealed a number of potential new molecular markers associated with *Sargassum* racks from the Caribbean. Even if further researches will have to address more specifically the intra and inter-sites variability as well as the variation of the communities depending on *Sargassum* morphotypes, we have identified here a number of potentially new or poorly described species, with some of these taxa performing selective functions for the host and/or involved in *Sargassum* degradation processes.

## Conclusion

Our study revealed specific micro- and meiofauna depending on the *Sargassum* samples. We also identified prokaryotic (archaea and bacteria) and eukaryotic biomarkers for marine waters at tide sites, near-shore and beach stranded wracks, and at inland storage sites. The description of this biodiversity provides a grounding for further investigations of the functions played by the epiphytes for the host, algal organic matter decomposition or nutrient transfers between trophic levels at both sea to land sites. Among other things, comparative and targeted investigations on biogeography and *Sargassum* holobionte composition, as well as the role of the degradation processes are required to further elucidate the environmental changes induced by *Sargassum* tides and their local consequences on biodiversity.

## Data Availability Statement

The datasets presented in this study can be found in online repositories. The names of the repository and accession numbers can be found in the article/Supplementary Material.

## Author Contributions

VH and PJL: conceptualization and investigations. VH, JL, MR-T, DAD, and PJL: sampling. VH, JL, and PJL: methodology and writing—original draft. PJL: funding acquisition. All authors contributed to the article and approved the submitted version.

## Conflict of Interest

The authors declare that the research was conducted in the absence of any commercial or financial relationships that could be construed as a potential conflict of interest.

## Publisher’s Note

All claims expressed in this article are solely those of the authors and do not necessarily represent those of their affiliated organizations, or those of the publisher, the editors and the reviewers. Any product that may be evaluated in this article, or claim that may be made by its manufacturer, is not guaranteed or endorsed by the publisher.
