# Supplementary material for: Sargassum Differentially Shapes the Microbiota Composition and Diversity at Coastal Tide Sites and Inland Storage Sites on Caribbean Islands
Source: Front Microbiol. 2021 Oct 29;12:701155. doi: 10.3389/fmicb.2021.701155 (PMC8586501; doi:10.3389/fmicb.2021.701155)
Supplement: Supplementary file 1 [file Data_Sheet_1.PDF]

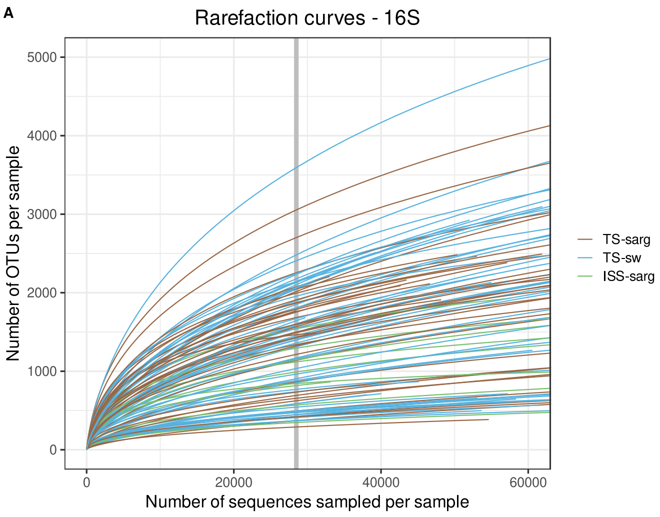


**Supplementary Figure S1: Rarefaction curves.** Rarefaction curves for the number of observed OTUs generated with (**A**) 16S rRNA gene amplicon datasets, (**B**) 18S rRNA gene amplicon datasets. The gray vertical lines correspond to the number of sequences used for the subsampling.
