# Supplementary material for: Sargassum Differentially Shapes the Microbiota Composition and Diversity at Coastal Tide Sites and Inland Storage Sites on Caribbean Islands
Source: Front Microbiol. 2021 Oct 29;12:701155. doi: 10.3389/fmicb.2021.701155 (PMC8586501; doi:10.3389/fmicb.2021.701155)
Supplement: Supplementary file 2 [file Data_Sheet_2.PDF]

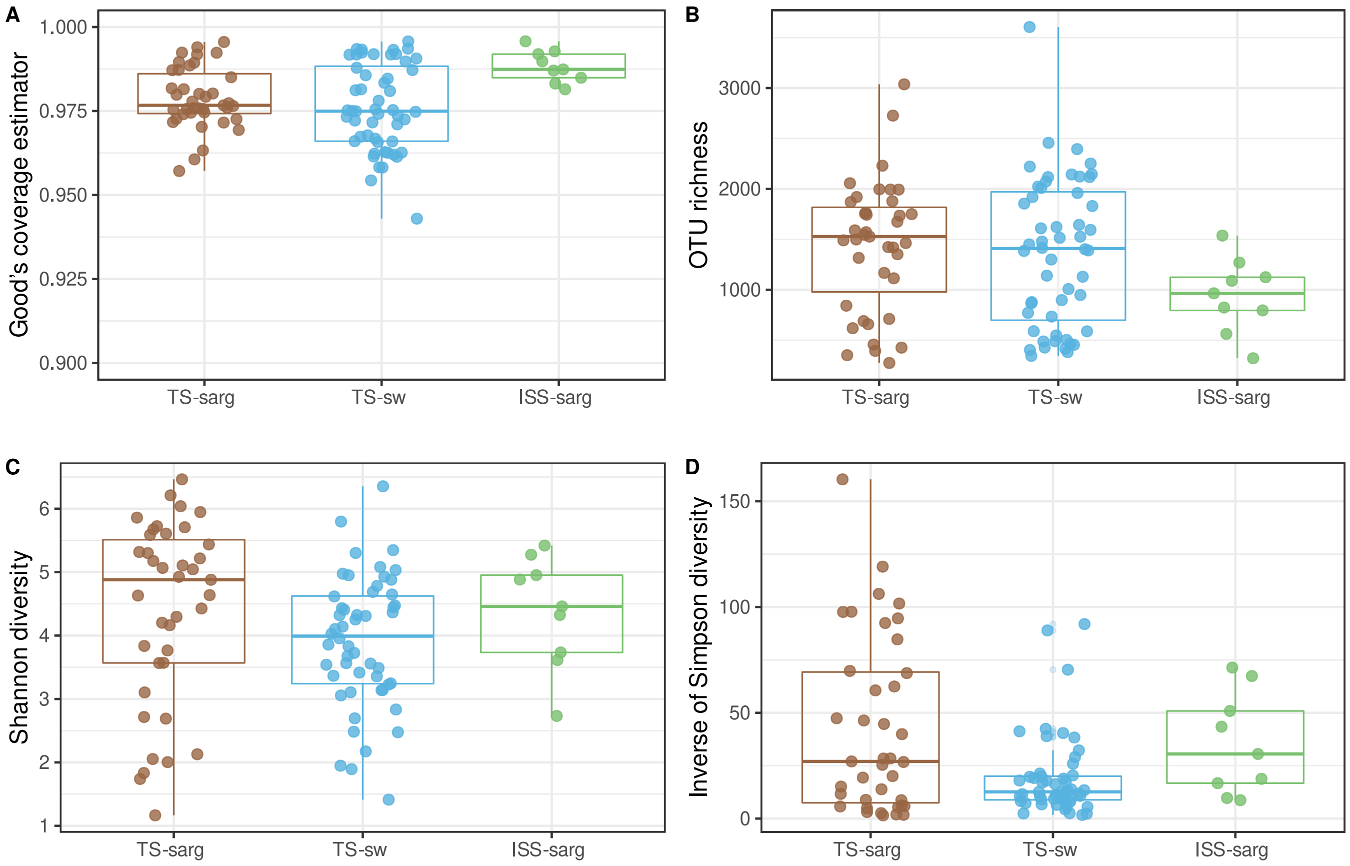

Supplementary Figure S2: Coverage estimation and *alpha* diversity indices for the 16S rRNA gene OTUs from seawater at tide sites (TS-sw), landing *Sargassum* (TS-sarg), *Sargassum* from inland storage sites (ISS-sarg) samples. (**A**) Good's coverage estimator. The *alpha* diversity of each sample was estimated using (**B**) the observed OTU richness, (**C**) the Shannon diversity index, and (**D**) the inverse of Simpson diversity index. To compare the *alpha* diversity indices between compartments, we used Kruskal-Wallis tests and corrected our P-values for multiple comparisons with the Benjamini-Hochberg method. No statistical differences were observed for any of the three *alpha* diversity indices.
