# Supplementary material for: Sargassum Differentially Shapes the Microbiota Composition and Diversity at Coastal Tide Sites and Inland Storage Sites on Caribbean Islands
Source: Front Microbiol. 2021 Oct 29;12:701155. doi: 10.3389/fmicb.2021.701155 (PMC8586501; doi:10.3389/fmicb.2021.701155)
Supplement: Supplementary file 4 [file Data_Sheet_4.PDF]

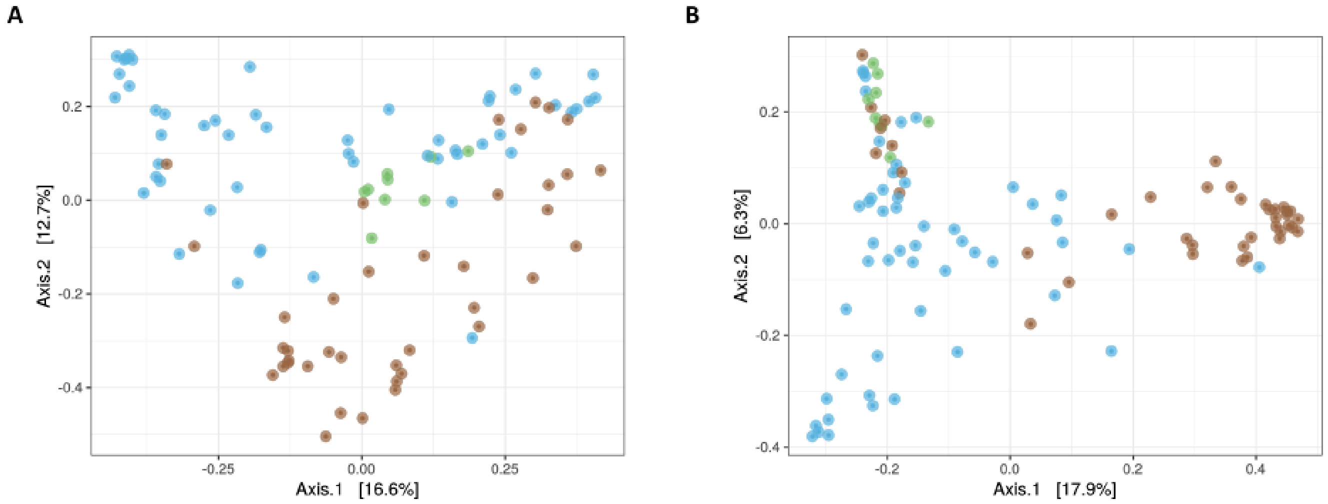

Supplementary Figure S4: Differences in community composition. Principal coordinates analyses (PCoA) of the microbial community composition based on Bray-Curtis dissimilarity matrices Indicate differences in community composition between seawater at tide sites (TS-sw, *blue*), *Sargassum* at tide sites *Sargassum* (TS-sarg, *brown*), and *Sargassum* at inland storage sites (ISS-sarg, *green*). (**A**) Prokaryotic community; and (**B**) Eukaryotic community.
