# Supplementary material for: Sargassum Differentially Shapes the Microbiota Composition and Diversity at Coastal Tide Sites and Inland Storage Sites on Caribbean Islands
Source: Front Microbiol. 2021 Oct 29;12:701155. doi: 10.3389/fmicb.2021.701155 (PMC8586501; doi:10.3389/fmicb.2021.701155)
Supplement: Supplementary file 5 [file Data_Sheet_5.PDF]

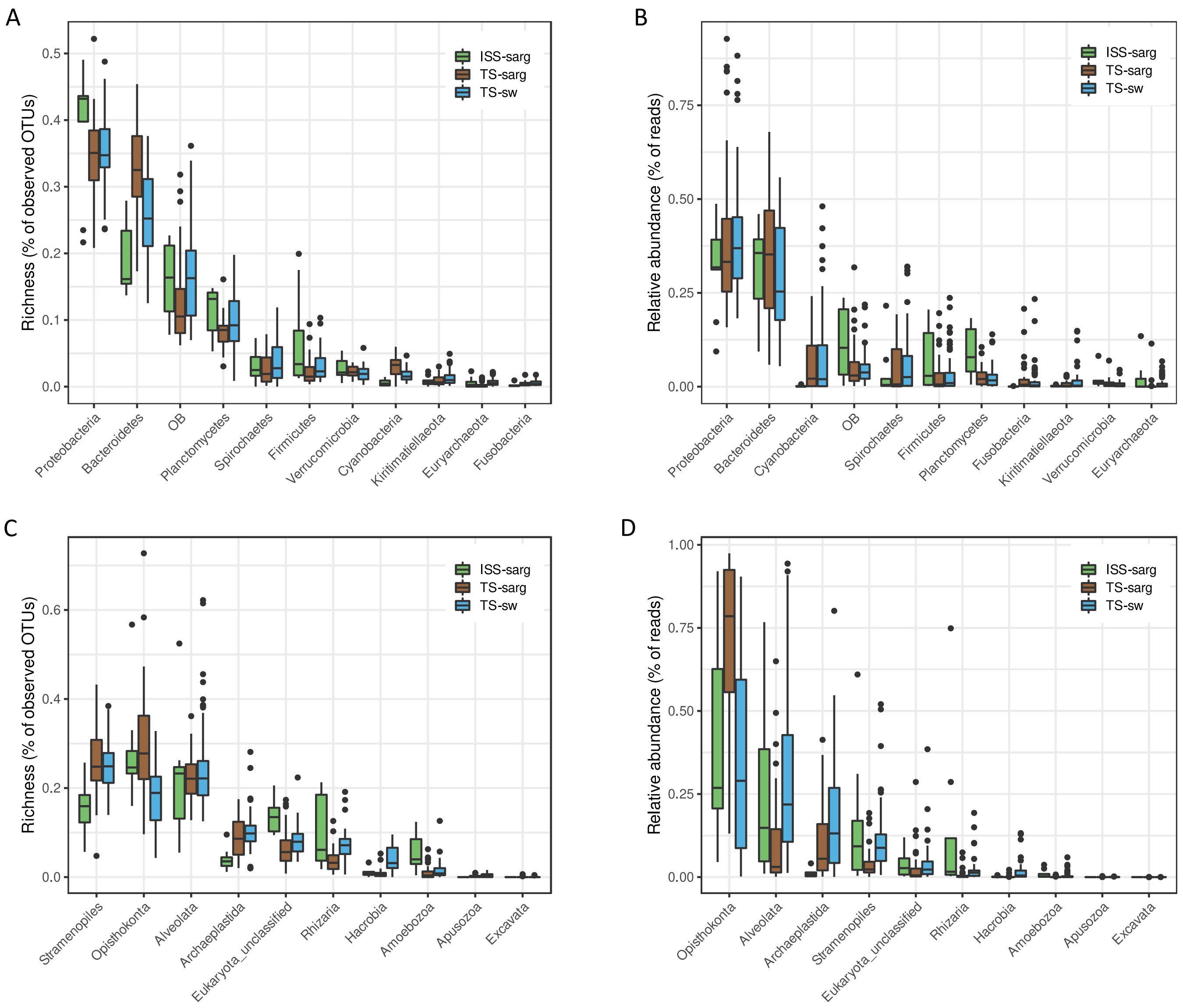

Supplementary Figure S5: Differences in the richness and relative abundance in the three compartments. Boxplots showing the distribution variations of the major clades within each compartment (**A**) based on the observed OTUs richness of the prokaryotic phyla (OB: other Bacteria), (**B**) based on the relative abundance of the prokaryotic phyla (OB: other Bacteria), (**C**) based on the observed OTUs richness of the main eukaryotic clades, and (**D**) based on the relative abundance of the main eukaryotic clades.
