# Supplementary material for: Sargassum Differentially Shapes the Microbiota Composition and Diversity at Coastal Tide Sites and Inland Storage Sites on Caribbean Islands
Source: Front Microbiol. 2021 Oct 29;12:701155. doi: 10.3389/fmicb.2021.701155 (PMC8586501; doi:10.3389/fmicb.2021.701155)
Supplement: Supplementary file 6 [file Data_Sheet_6.PDF]

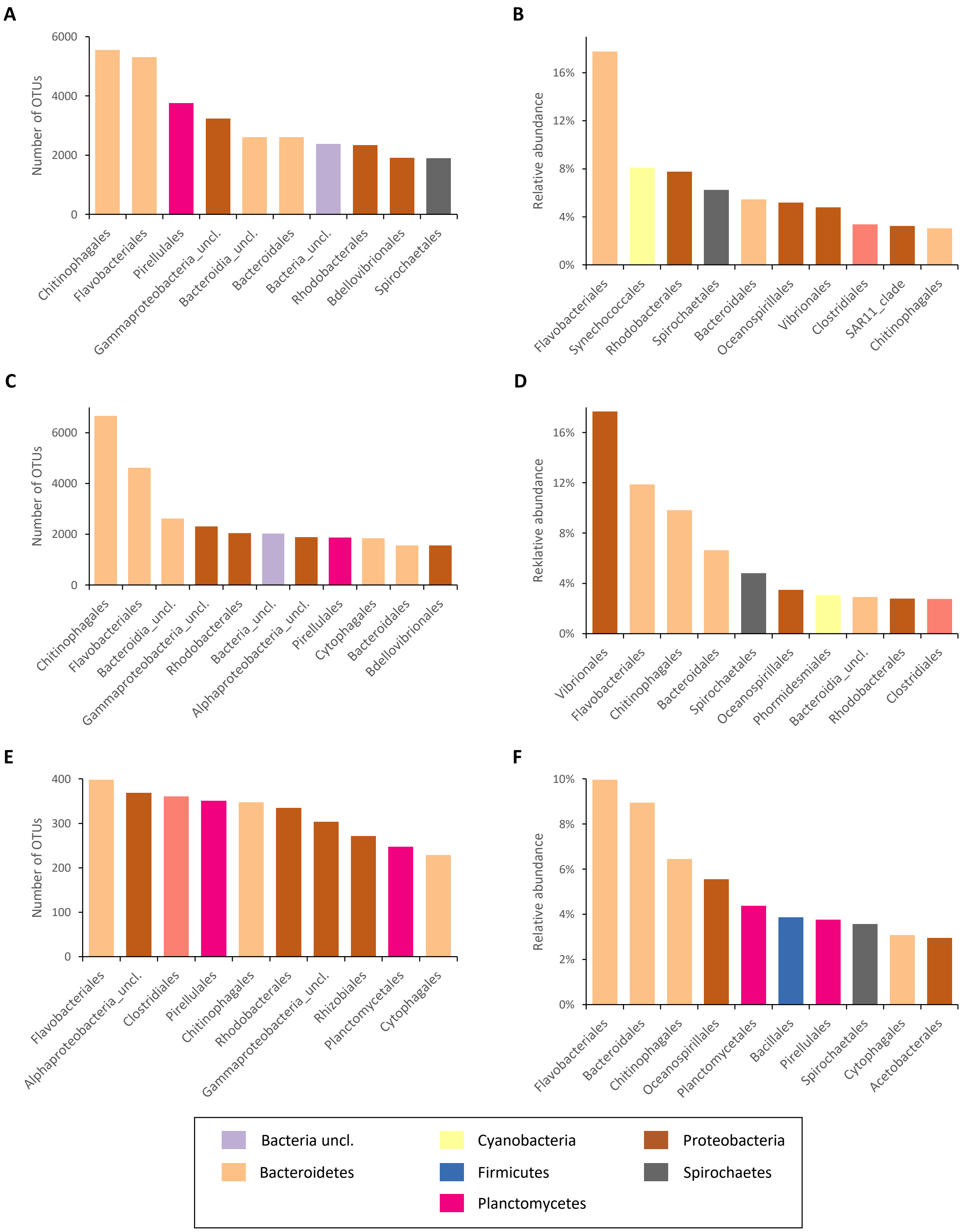

Supplementary Figure S6: Prokaryotic composition of the most rich or abundant orders for the three compartments. Observed OTU richness (**A**) and relative abundance (**B**) for seawater at tide sites (TS-sw). Observed OTU richness (**C**) and relative abundance (**D**) for landing *Sargassum* (TS-sarg). Observed OTU richness (**E**) and relative abundance (**F**) associated to *Sargassum* from inland storage sites (ISS-sarg). The relative abundance is given as percent of the relative abundance per compartment. Uncl. corresponds to unclassified.
