# Supplementary material for: Sargassum Differentially Shapes the Microbiota Composition and Diversity at Coastal Tide Sites and Inland Storage Sites on Caribbean Islands
Source: Front Microbiol. 2021 Oct 29;12:701155. doi: 10.3389/fmicb.2021.701155 (PMC8586501; doi:10.3389/fmicb.2021.701155)
Supplement: Supplementary file 8 [file Data_Sheet_8.PDF]

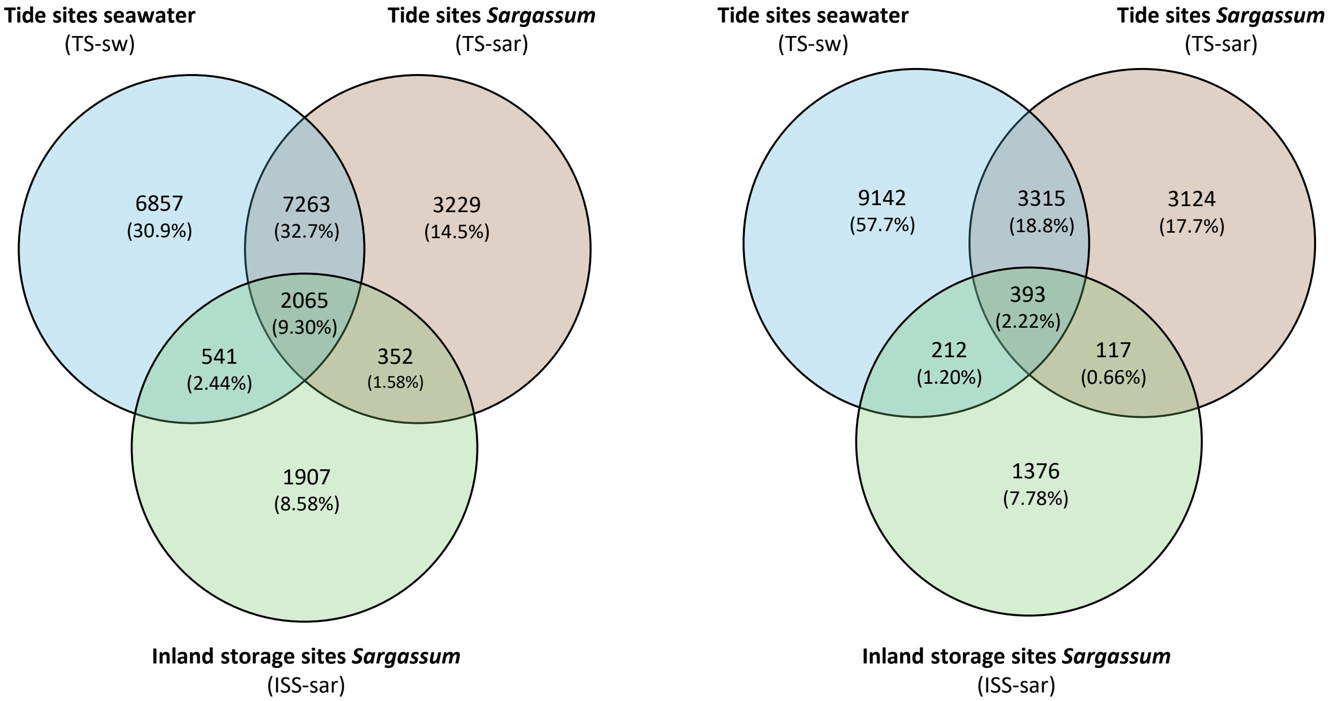

Supplementary Figure S8: Venn diagrams showing the specific and shared OTUs between tide sites seawater, tide sites *Sargassum*, and inland storage sites *Sargassum* from Guadeloupe and Martinique. Prokaryotes (*left*) and Eukaryotes (*right*).
