# Supplementary material for: Sargassum Differentially Shapes the Microbiota Composition and Diversity at Coastal Tide Sites and Inland Storage Sites on Caribbean Islands
Source: Front Microbiol. 2021 Oct 29;12:701155. doi: 10.3389/fmicb.2021.701155 (PMC8586501; doi:10.3389/fmicb.2021.701155)
Supplement: Supplementary file 10 [file Data_Sheet_10.PDF]

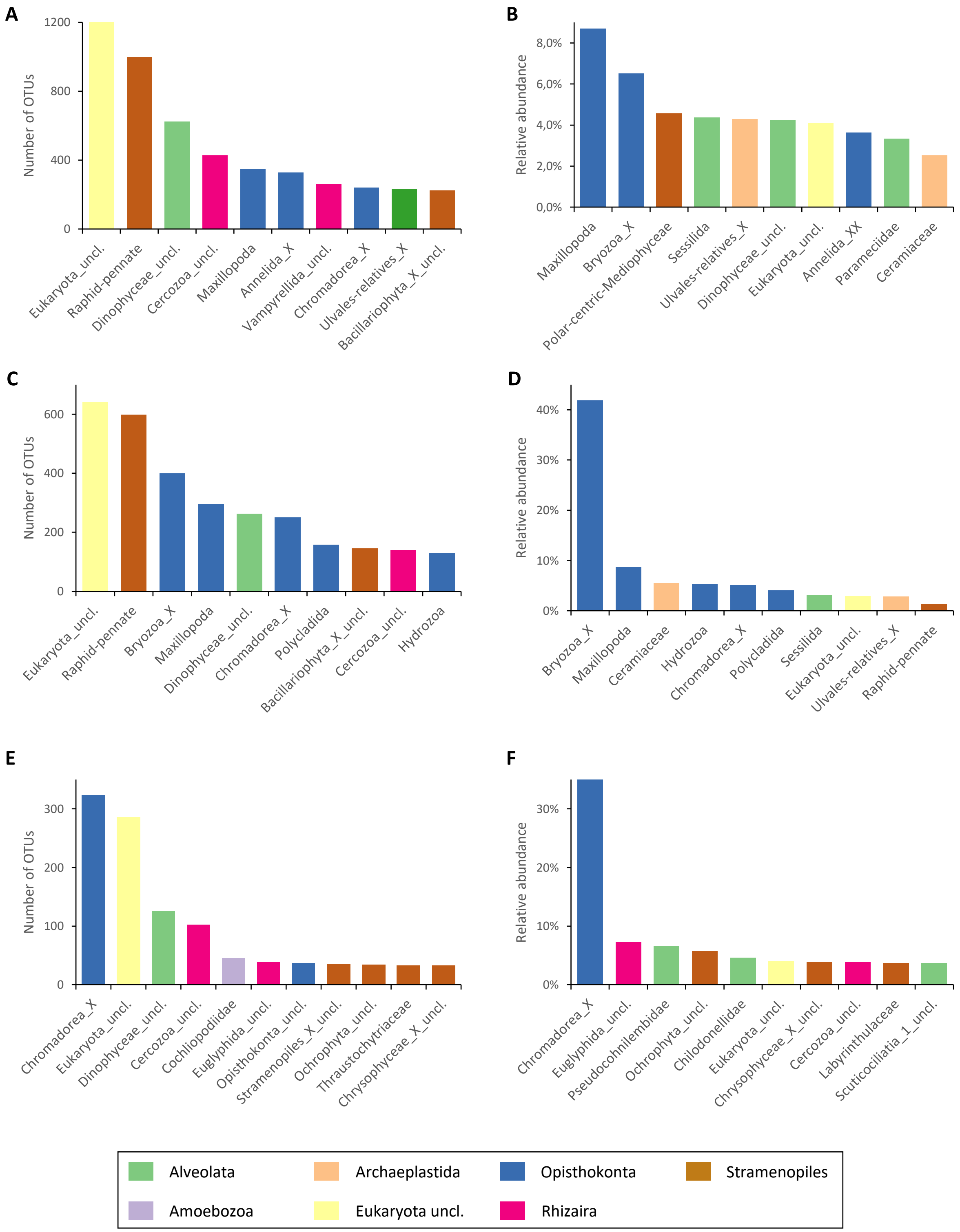

Supplementary Figure S10: Differences in the most rich and abundant clades (rank 5 based on PR2) for the three compartments. Observed OTU richness (**A**) and relative abundance (**B**) of seawater at tide sites (TS-sw). Observed OTU richness (**C**) and relative abundance (**D**) of landing *Sargassum* (TS-sarg). Observed OTU richness (**E**) and relative abundance (**F**) associated to *Sargassum* from terrestrial storage sites (ISS-sarg). The relative abundance is given as percent of the relative abundance per compartment. Uncl. corresponds to unclassified.
