# Supplementary material for: Sargassum Differentially Shapes the Microbiota Composition and Diversity at Coastal Tide Sites and Inland Storage Sites on Caribbean Islands
Source: Front Microbiol. 2021 Oct 29;12:701155. doi: 10.3389/fmicb.2021.701155 (PMC8586501; doi:10.3389/fmicb.2021.701155)
Supplement: Supplementary file 11 [file Data_Sheet_11.PDF]

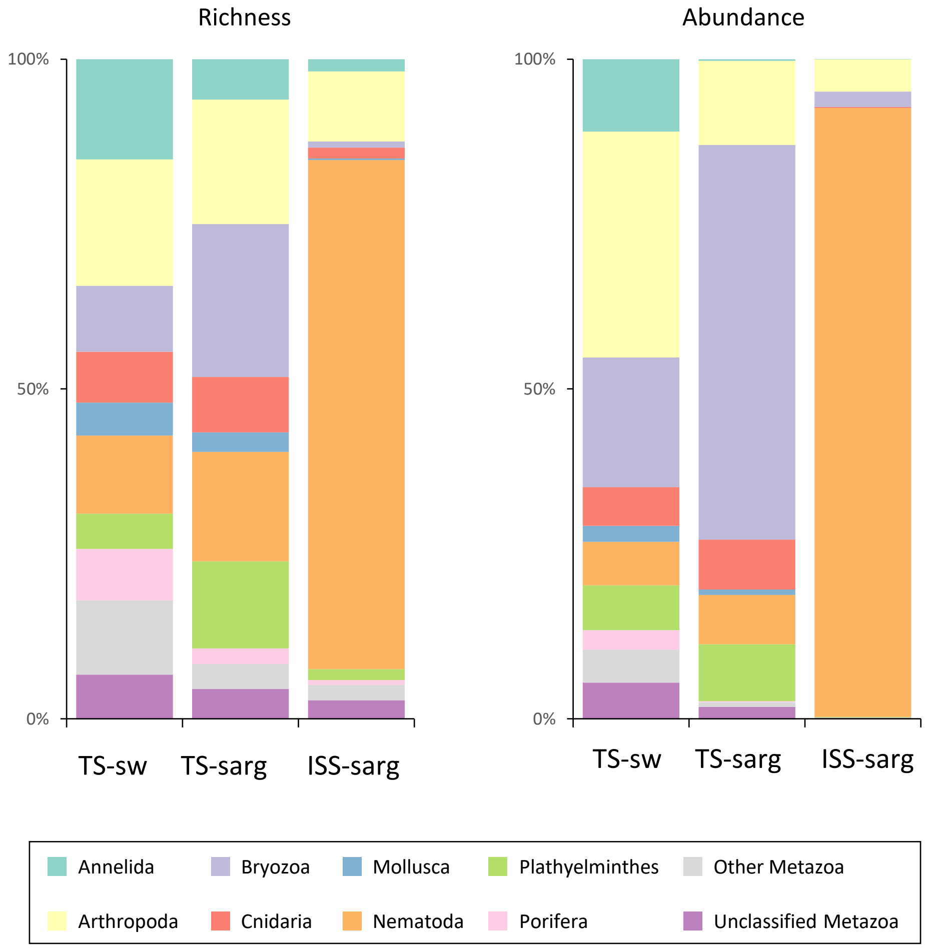

Supplementary Figure S11: Richness and relative abundance within the Metazoa. The observed OTU richness is given as percent of total richness per compartment. The abundance is given as percent of the relative abundance per compartment.
