# Supplementary material for: Sargassum Differentially Shapes the Microbiota Composition and Diversity at Coastal Tide Sites and Inland Storage Sites on Caribbean Islands
Source: Front Microbiol. 2021 Oct 29;12:701155. doi: 10.3389/fmicb.2021.701155 (PMC8586501; doi:10.3389/fmicb.2021.701155)
Supplement: Supplementary file 12 [file Data_Sheet_12.PDF]

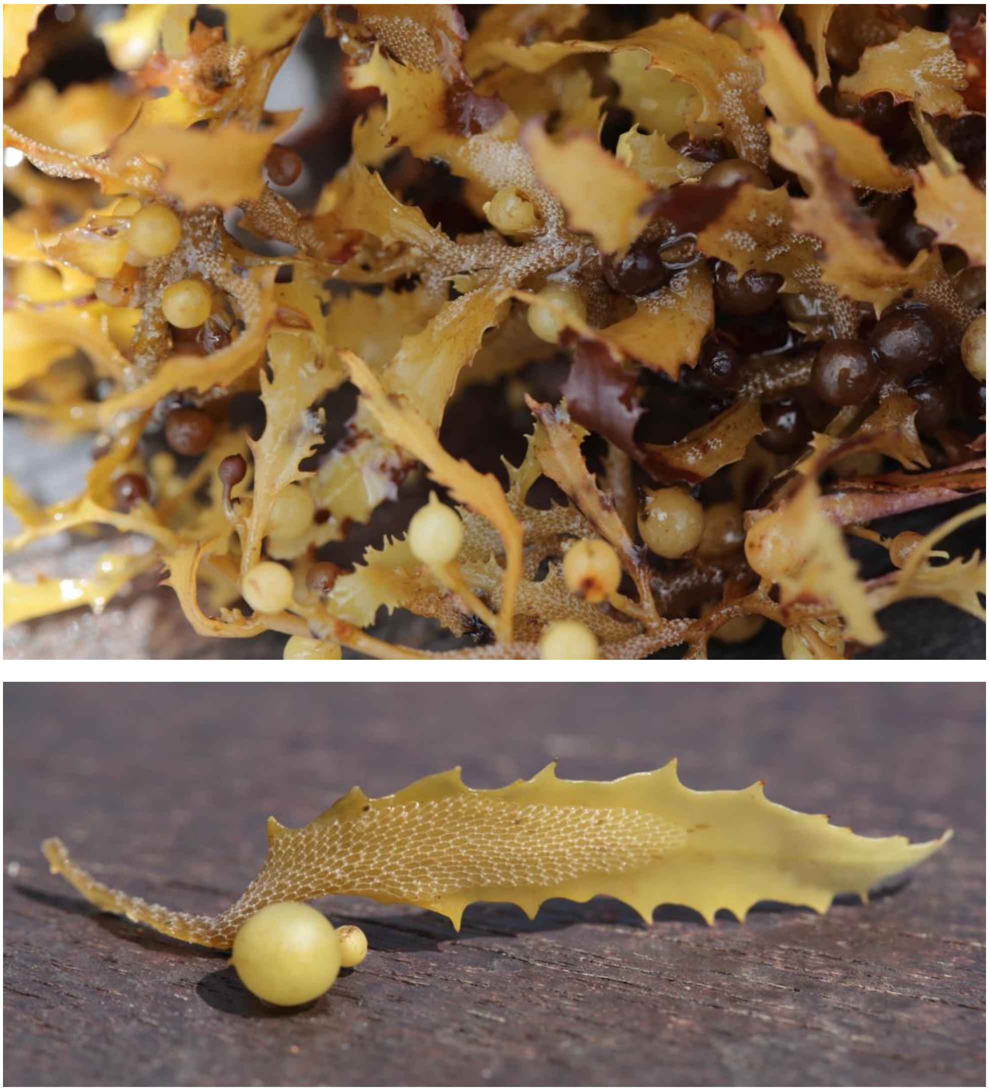

Supplementary Figure S12: Images showing Bryozoa on *Sargassum*. This epiphytic bryozoan forms encrusting, lacy mat-like colonies of very small, rectangular autozooids found on stem, leaf and gas bladders.
